# HSP90 Inhibitors, Geldanamycin and Radicicol, Enhance Fisetin-Induced Cytotoxicity via Induction of Apoptosis in Human Colonic Cancer Cells

**DOI:** 10.1155/2013/987612

**Published:** 2013-06-11

**Authors:** Ming-Shun Wu, Gi-Shih Lien, Shing-Chuan Shen, Liang-Yo Yang, Yen-Chou Chen

**Affiliations:** ^1^Graduate Institute of Clinical Medicine, Taipei Medical University, Taipei 110, Taiwan; ^2^Division of Gastroenterology, Department of Internal Medicine, Wan Fang Hospital, Taipei Medical University, Taipei 116, Taiwan; ^3^Graduate Institute of Medical Sciences, Taipei Medical University, Taipei 110, Taiwan; ^4^Department of Physiology, Graduate Institute of Neuroscience, Taipei Medical University, Taipei 110, Taiwan; ^5^Cancer Research Center and Orthopedics Research Center, Taipei Medical University Hospital, Taipei 110, Taiwan

## Abstract

We revealed the cytotoxic effect of the flavonoid, fisetin (FIS), on human COLO205 colon cancer cells in the presence and absence of the HSP90 inhibitors, geldanamycin (GA) and radicicol (RAD). Compared to FIS treatment alone of COLO205 cells, GA and RAD significantly enhanced FIS-induced cytotoxicity, increased expression of cleaved caspase-3 and the PAPR protein, and produced a greater density of DNA ladder formation. GA and RAD also reduced the MMPs with induction of caspase-9 protein cleavage in FIS-treated COLO205 cells. Increased caspase-3 and -9 activities were detected in COLO205 cells treated with FIS+GA or FIS+RAD, and the intensity of DNA ladder formation induced by FIS+GA was reduced by adding the caspase-3 inhibitor, DEVD-FMK. A decrease in Bcl-2 but not Bcl-XL or Bax protein by FIS+GA or FIS+RAD was identified in COLO205 cells by Western blotting. A reduction in p53 protein with increased ubiquitin-tagged proteins was observed in COLO205 cells treated with FIS+GA or FIS+RAD. Furthermore, GA and RAD reduced the stability of the p53 protein in COLO205 cells under FIS stimulation. The evidence supports HSP90 inhibitors possibly sensitizing human colon cancer cells to FIS-induced apoptosis, and treating colon cancer by combining HSP90 inhibitors with FIS deserves further in vivo study.

## 1. Introduction

Colorectal cancer is one of the leading causes of cancer deaths in western countries and has become a common malignancy in Asia due to significant changes in diet and lifestyle over the past few decades. A combination of surgery, chemotherapy, and targeted therapy is the mainstream anticancer therapy; however, there are possible systemic toxic effects with such chemical compounds. In contrast, flavonoids are natural dietary compounds. Epidemiologic studies showed that high intake of flavonoid-enriched vegetables and fruits can reduce the risk of colon cancer [1]. In vitro studies revealed that flavonoids can reduce the risk of colon cancer via their chemopreventive properties including induction of cell-cycle arrest and apoptosis and their antiproliferative effect, thus providing a novel therapeutic option for cancer treatment by complementary and alternative medicine [2].

Fisetin (3,7,3,4-tetrahydroxyflavone; FIS) is one of the major flavonoids widely found in fruits and vegetables including apples, onions, grapes, and cucumbers and was shown to exert a variety of biological activities, including antioxidant, anti-inflammatory, anti-invasive, and antiproliferative effects. FIS was reported to inhibit the proliferation of several cancer cells, including hepatocellular carcinoma, prostate cancer, and colon cancer cells. Additionally, FIS may also act as an inhibitor of cyclin-dependent kinases (CDKs) to induce cell-cycle arrest in cancer cells [3]. Furthermore, FIS was reported to induce apoptosis in different cancer cells, including hepatocellular carcinoma SK-HEP-1, myeloleukemic HL-60, and prostate cancer LNCaP cells [4–6]. Recent studies showed that FIS was able to induce apoptosis in both p53-wild-type and p53-mutant colon cancer cells [7, 8]. These accumulating results provide evidence of the potent anticancer activity of FIS; however, the effects of FIS on apoptosis of colon cancer cells and its underlying mechanisms have yet to be clearly elucidated.

Heat shock protein 90 (HSP90) is an essential chaperon for integrity and function of a wide range of oncogenic client proteins which are implicated in carcinogenesis [9]. Compared to normal tissues, HSP90 is generally overexpressed in tumors by around 2~10-fold and is associated with a poor prognosis [10–12]. Through its ability to control the stability and activity of client proteins involved in the oncogenic process, targeting HSP90 has the potential to affect most of the hallmarks of cancer. Since the first HSP90 inhibitor, geldanamycin (GA), was found in 1994, HSP90 inhibitors have emerged as a promising therapeutic intervention for a wide variety of human cancers in the past two decades [13]. Nowadays, colorectal cancer expressing epidermal growth factor receptor (EGFR), a client protein of HSP90, has been targeted using tyrosine kinase inhibitors and monoclonal antibodies [14].

In this study, we investigated the role of HSP90 inhibitors in the anticancer effects of FIS against human colon cancer cells. The HSP90 inhibitors, geldanamycin (GA) and radicicol (RAD), effectively enhanced the cytotoxicity of human COLO205 colon cancer cells under FIS stimulation. GA and RAD induced apoptosis of FIS-treated COLO205 cells through disruption of the mitochondrial membrane potential (MMP) and decreased Bcl-2 and p53 protein expressions via enhancement of protein ubiquitination and reduction of p53 protein stability. Our results provide a molecular basis for treating human colon cancer with FIS and HSP90 inhibitors.

## 2. Materials and Methods

### 2.1. Cell Culture

COLO205 colonic carcinoma cells were obtained from the American Type Culture Collection (ATCC, Manassas, VA, USA). Cells were maintained in RPMI 1640 supplemented with antibiotics (100 U/mL penicillin A and 100 U/mL streptomycin) and 10% heat-inactivated fetal bovine serum (FBS; Gibco/BRL, Grand Island, NY, USA) and maintained in a 37°C humidified incubator containing 5% CO_2_.

### 2.2. Agents

The chemical reagents of FIS, GA, RAD, BCIP, MTT, and nitroblue tetrazolium (NBT) were obtained from Sigma Chemical (St. Louis, MO, USA). Antibodies of *α*-tubulin, poly(ADP-ribose)polymerase (PARP), caspase-3, caspase-9, Bcl-2, and Bax were from Santa Cruz Biotechnology (Santa Cruz, CA, USA). The colorigenic synthetic peptide substrates, Ac-DEVD-pNA (caspase-3 substrate) and Ac-YVAD-pNA (caspase-9 substrate), and the protease inhibitor for caspase-3 (Ac-DEVD-FMK) were purchased from Calbiochem (Darmstadt, Germany).

### 2.3. Cell Viability Assay

Cell viability was assessed by MTT staining. Briefly, cells were plated at a density of 10^5^ cells/well into 24-well plates. At the end of treatment, the supernatant was removed, and 30 *μ*L of the tetrazolium compound, MTT, and 270 mL of fresh RPMI medium were added. After incubation for 4 h at 37°C, 200 *μ*L of 0.1 N HCl in 2-propanol was placed in each well to dissolve the tetrazolium crystals. Finally, the absorbance at a wavelength of 600 nm was recorded using an enzyme-linked immunosorbent assay (ELISA) plate reader (Molecular Devices, MA, USA).

### 2.4. Western Blotting

Total cellular extracts (30 *μ*g) were prepared and separated on 8% sodium dodecylsulfate (SDS)-polyacrylamide minigels for PARP detection and 12% SDS-polyacrylamide minigels for caspase-3, caspase-9, the Bcl-2 family, tERK, pERK, and *α*-tubulin detection and transferred to Immobilon polyvinylidene difluoride membranes (Millipore, Bedford, MA, USA). Membranes were incubated at 4°C with 1% bovine serum albumin and then incubated with the indicated antibodies for further 3 h at room temperature followed by incubation with an alkaline phosphatase-conjugated immunoglobulin G (IgG) antibody for 1 h. Protein was visualized by incubating with the colorimetric substrates, NBT and BCIP.

### 2.5. DNA Fragmentation Assay

Colonic carcinoma cells under different treatments were collected and then lysed in 100 *μ*L of lysis buffer (50 mM Tris at pH 8.0, 10 mM ethylenediaminetetraacetic acid (EDTA), 0.5% sodium sarcosinate, and 1 mg/mL proteinase K) for 3 h at 56°C. Then, 0.5 mg/mL RNase A was added to each reaction for another 1 h at 56°C. DNA was extracted with phenol/chloroform/isoamyl alcohol (25/24/1) before loading. Then, DNA samples were mixed with 6 *μ*L of loading buffer (50 mM Tris, 10 mM EDTA, 1% (w/w) glycerol, and 0.025% (w/w) bromophenol blue) and loaded onto a 2% agarose gel containing 0.1 mg/mL ethidium bromide. The agarose gels were run at 100 V for 45 min in TBE buffer then observed and photographed under UV light.

### 2.6. Measurement of the MMP

After different treatments, cells were incubated with 40 nM DiOC6(3) for 15 min at 37°C then washed with ice-cold phosphate-buffered saline (PBS) and collected by centrifugation at 500 ×g for 10 min. Collected cells were resuspended in 500 *µ*L of PBS containing 40 nM DiOC6(3). Fluorescence intensities of DiOC6(3) were analyzed on a flow cytometer (FACScan, Becton Dickinson) with respective excitation and emission settings of 484 and 500 nm.

### 2.7. Analysis of Caspase-3 and -9 Activities

Ac-DEVD-pNA was used as a colorimetric protease substrate to detect caspase-3 activity. After different treatments, cells were collected and washed three times with PBS and resuspended in 50 mM Tris-HCl (pH 7.4), 1 mM EDTA, and 10 mM EGTA. Cell lysates were clarified by centrifugation at 15,000 rpm for 3 min, and clear lysates containing 200 *μ*g of protein were incubated with 100 mM of the indicated specific colorimetric substrates at 37°C for 1 h. Alternative activities of caspase-3 and -9 enzymes were described by the cleavage of a colorimetric substrate and measuring the absorbance at 405 nm.

### 2.8. Statistical Analysis

Values are expressed as the mean ± standard error (SE) of triplicate experiments. The significance of the difference from the respective controls for each experiment was assayed using a one-way analysis of variance (ANOVA) for a post-hoc Bonferroni analysis when applicable, and *P* values of <0.05 or <0.01 were considered statistically significant.

## 3. Results

### 3.1. HSP90 Inhibitors, GA and RAD, Enhanced FIS-Induced Cytotoxicity via Inducing Apoptosis in COLO205 Cells

**Figure 1:**
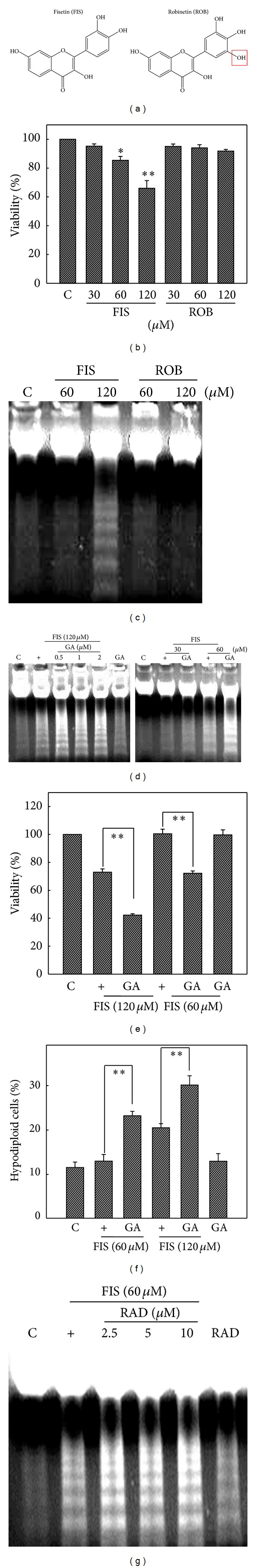
Effects of geldanamycin (GA) and radicicol (RAD) on the viability of COLO205 colorectal carcinoma cells under fisetin (FIS) stimulation. (a) The chemical structures of FIS and the structurally related robinetin (ROB) are depicted. (b) FIS reduction of cell viability of COLO205 cells. COLO205 cells were treated with the indicated concentrations (30, 60, and 120 *μ*M) of FIS for 24 h, and cell viability was examined by an MTT assay. (c) DNA ladders induced by FIS or ROB (60 or 120 *μ*M) in COLO205 cells were detected by agarose electrophoresis. (d) GA enhanced DNA ladder formation in COLO205 cells stimulated by FIS. (Left panel) cells were treated with different concentrations of GA (0.5, 1, and 2 *μ*M) with or without FIS (120 *μ*M) stimulation for 24 h. (Right panel) cells were treated with GA (2 *μ*M) with or without FIS (30 or 60 *μ*M) stimulation for 24 h. The integrity of DNA was analyzed by agarose electrophoresis. (e) GA increased the cytotoxicity of FIS against the viability of COLO205 cells. Cells were treated with GA (2 *μ*M) and FIS (60 or 120 *μ*M) for 24 h, and the viability of cells was examined by an MTT assay. (f) Increases in the percentage of hypodiploid cells by GA in FIS-treated COLO205 cells. As described above, the ratio of hypodiploid cells under different treatments was examined by a flow cytometric analysis using propidium iodide (PI) staining. (g) RAD addition increased the intensity of DNA ladder formation in FIS-treated COLOL205 cells. Cells were treated with different concentrations (2.5, 5, and 10 *μ*M) of RAD with FIS (60 *μ*M) for 24 h, and the integrity of DNA was analyzed. Each data point was calculated from three triplicate groups, and data are displayed as the mean ± S.E. *P < 0.05 and **P < 0.01 denote a significant difference compared to the control (C) group (Figure 1(b)) or between the indicated groups (Figures 1(e) and 1(f)).

The chemical structures of FIS and its structurally related compound, robinetin (ROB), are depicted in Figure 1(a); ROB contains an additional OH group at C5 of FIS. Data of the MTT assay showed that FIS but not ROB at concentrations of 60 and 120 *μ*M showed slight but significant reductions in the viability of COLO205 colon carcinoma cells by 11.7% ± 3.2% and 27.6% ± 4.7%, respectively (Figure 1(b)). Analysis of DNA integrity of COLO205 cells under FIS or ROB treatment showed that FIS at a concentration of 120 *μ*M was able to reduce the integrity of DNA via increased DNA ladder formation in COLO205 cells according to DNA agarose electrophoresis (Figure 1(c)). In the presence of the HSP90 inhibitor, GA, an increase in DNA ladder intensity was observed in FIS-treated COLO205 cells (Figure 1(d)). Data of the MTT assay showed that the addition of GA significantly enhanced COLO205 cell death by FIS (Figure 1(e)). A significant increase in the ratio of hypodiploid cells (sub-G1) with GA was detected in FIS-treated COLO205 cells by a flow cytometric analysis (Figure 1(f)). Increased intensity of DNA ladders by another HSP90 inhibitor, RAD, was observed in COLO205 cells under FIS stimulation (Figure 1(g)). These data support that application of HSP90 inhibitors, such as GA and RAD, may potentiate the cytotoxic effect of FIS against the viability of COLO205 cells.

### 3.2. Induction of Caspase-3-Mediated Apoptosis in GA- or RAD-Enhanced FIS-Induced COLO205 Cell Apoptosis

**Figure 2:**
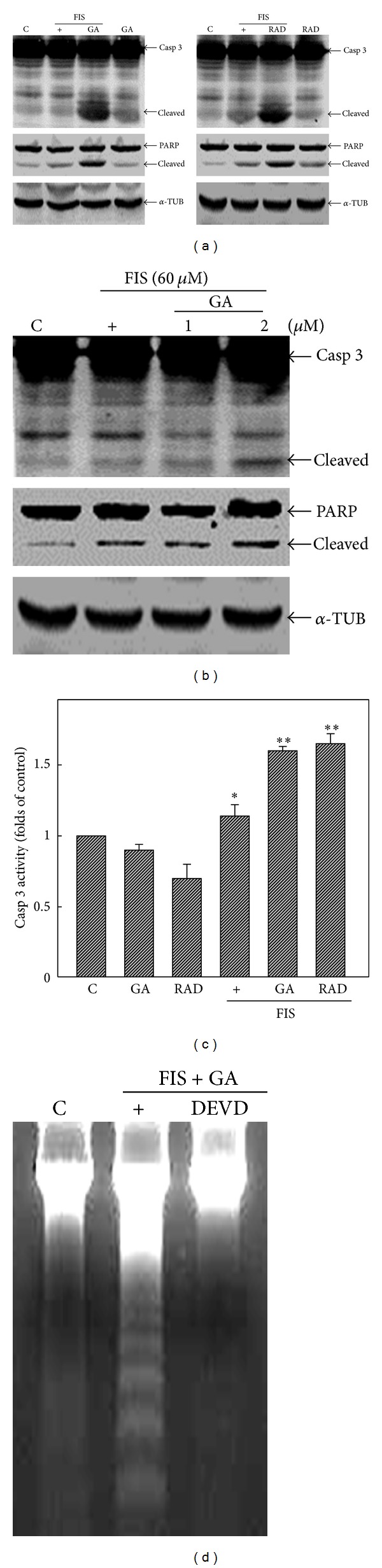
Geldanamycin (GA) and radicicol (RAD) increased caspase-3 and PARP protein cleavage in fisetin- (FIS-) treated COLO205 cells. (a) GA and RAD induced cleavage of caspase-3 and PARP in FIS-treated COLOL205 cells. COLO205 cells were treated with GA (2 *μ*M) or RAD (5 *μ*M) with or without FIS (60 *μ*M) for 24 h, and the expressions of caspase-3, PARP, and *α*-tubulin protein were detected by western blotting using specific antibodies. (b) Concentration-dependent investigation of GA on caspase-3 and PARP protein cleavage in FIS-treated COLO205 cells. (c) Effects of GA (2 *μ*M), RAD (5 *μ*M), FIS (60 *μ*M), FIS (60 *μ*M)+GA (2 *μ*M), and FIS (60 *μ*M)+RAD (5 *μ*M) on caspase-3 enzyme activity were examined using Ac-DEVD-pNA as a caspase-3-specific peptidyl substrate. Cells were treated with the indicated components for 24 h, and caspase-3 activity was measured using Ac-DEVD-pNA as a substrate. (d) The addition of the caspase-3 peptidyl inhibitor, Ac-DEVD-FMK, inhibited FIS+GA-induced DNA ladder formation in COLO205 cells. Cells were incubated with the caspase-3 inhibitor, Ac-DEVD-FMK, for 2 h, followed by FIS+GA stimulation for additional 24 h, and DNA integrity was analyzed. Each data point was calculated from three triplicate groups, and data are displayed as the mean ± S.E. *P < 0.05 and **P < 0.01 denote a significant difference from the control (C) group.

We further investigated if activation of caspase-3 was involved in GA- or RAD-enhanced apoptosis of COLO205 cells under FIS stimulation. As shown in Figure 2(a), increases in cleaved caspase-3 and a caspase-3 substrate PARP protein were detected in GA- or RAD-treated COLO205 cells under FIS stimulation. No change in the expression of *α*-tubulin (*α*-TUB) was examined as an internal control to verify that similar amounts of protein were loaded in each lane. Additionally, GA at a concentration of 2 *μ*M significantly increased caspase-3 and PARP cleavage in FIS-treated COLO205 cells (Figure 2(b)). FIS alone showed a slight but significant increase in caspase-3 activity, and a more significant increase in caspase-3 activity was detected in FIS+GA- or FIS+RAD-treated COLO205 cells using Ac-DEVD-pNA as a specific caspase-3 fluorescent substrate (Figure 2(c)). Data of DNA analysis showed that the addition of the peptidyl caspase-3 inhibitor, Ac-DEVD-FMK, inhibited FIS+GA-induced DNA ladder formation in COLO205 cells (Figure 2(d)). This indicated that GA- or RAD-enhanced FIS-induced cell death was mediated by activation of a caspase-3-mediated apoptotic pathway.

### 3.3. Loss of the MMP with Activation of Caspase-9 in GA- or RAD-Enhanced FIS-Induced COLO205 Cell Apoptosis

**Figure 3:**
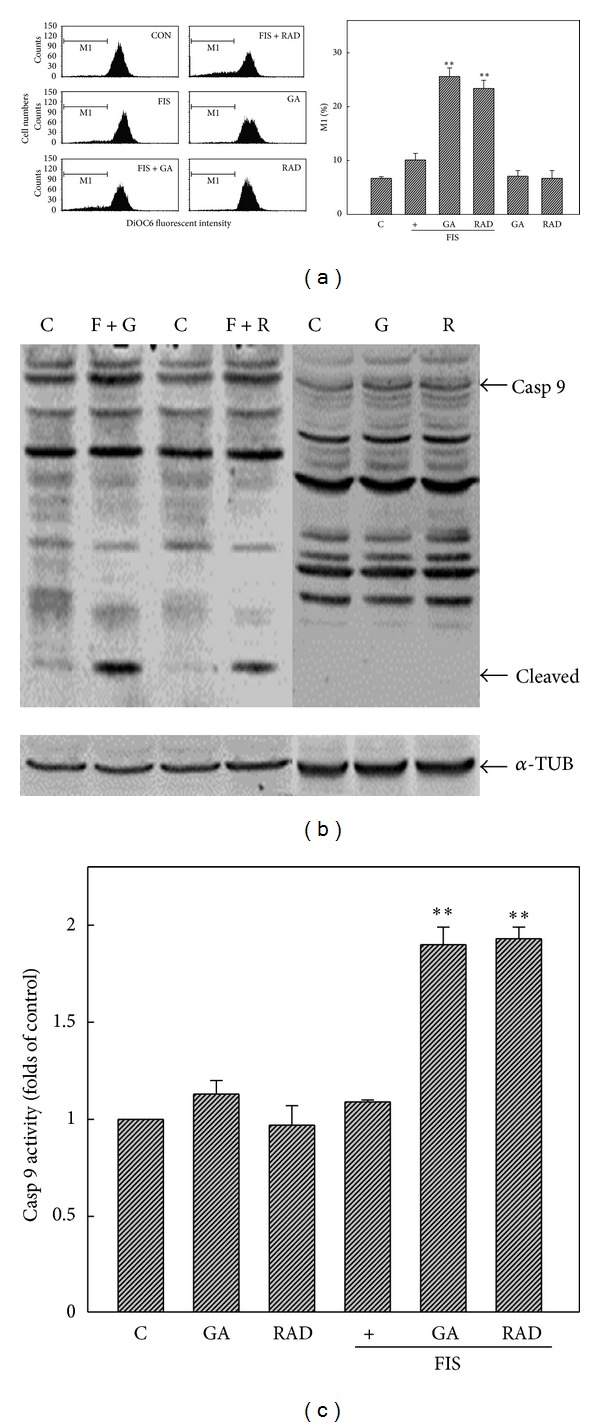
Disruption of the mitochondrial membrane potential (MMP) by fisetin (FIS)+geldanamycin (GA) or FIS+radicicol (RAD) in COLO205 cells. (a) GA or RAD increased loss of the MMP by FIS in COLO205 cells. Cells were treated with GA (G; 2 *μ*M) or RAD (R; 5 *μ*M) with or without FIS (F; 60 *μ*M) for 6 h, and the MMP was detected by a flow cytometric analysis using DiOC6 as a fluorescent dye. (Left panel) a representative of flow cytometric analysis. (Right panel) percentage of M1 was measured and expressed as the mean ± S.E. from three independent experiments. (b) GA or RAD induced cleavage of the caspase-9 protein in FIS-treated COLO205 cells. Cells were treated with GA, RAD, FIS+GA, or FIS+RAD for 24 h, and expression of caspase-9 (Casp 9) protein was detected by western blotting. (c) Increased caspase-9 activity by FIS+GA or FIS+RAD in COLO205 cells. The peptidyl caspase 9 substrate, Ac-YVAD-pNA, was used to detect caspase-9 activity in COLO205 cells under different treatments. Each data point was calculated from three triplicate groups, and data are displayed as the mean ± S.E. **P < 0.01 denotes a significant difference from the control (C) group.

**Figure 4:**
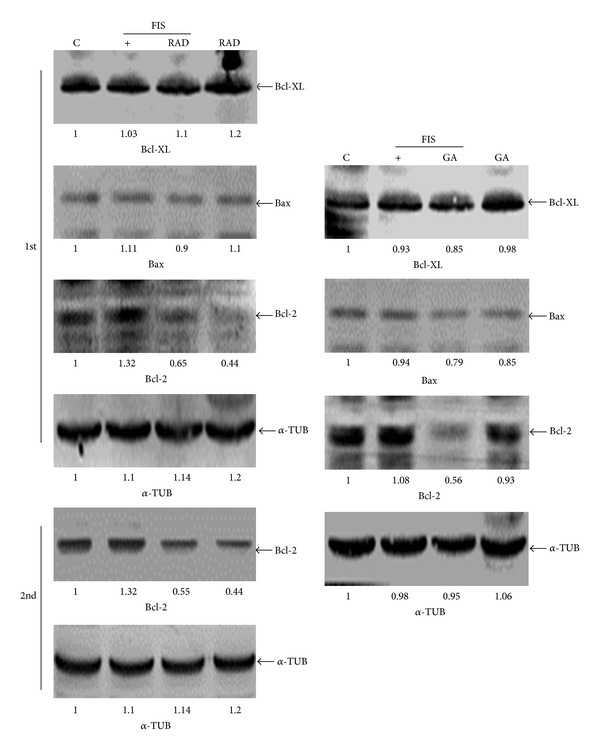
Alternative expression of Bcl-2 family proteins including Bcl-XL, Bcl-2, and Bax in fisetin- (FIS-), geldanamycin- (GA-), and radicicol- (RAD-) treated COLO205 cells. COLO205 cells were treated with FIS (60 *μ*M), GA (2 *μ*M), RAD (5 *μ*M), or their combinations for 24 h, and expressions of the indicated proteins were detected by Western blotting using specific antibodies. Data were repeated at least three times, and similar results were obtained. Data related to RAD-inhibited Bcl-2 protein expression from two independent experiments as labeled in 1st and 2nd were involved. The intensity of each band was examined by a densitometric analysis (ImageJ) and expressed as multiples of the control.

We further studied if GA- or RAD-enhanced apoptosis of FIS-treated COLO205 cells occurred through destroying mitochondrial homeostasis. The MMP was detected by DiOC6(3) staining, and results in Figure 3(a) show that FIS treatment did not affect the MMP, and a significant decrease in the MMP was detected in FIS+GA- and FIS+RAD-treated COLO205 cells, represented here as a decrease in the fluorescence intensity. GA and RAD alone showed no effect on the MMP in COLO205 cells. Additionally, induction of cleavage of the caspase-9 protein by FIS+GA or FIS+RAD was detected in COLO205 cells by Western blotting (Figure 3(b)). Measurement of caspase-9 activity using the specific fluorescent caspase-9 substrate, Ac-ZEVD-pNA, showed that the addition of GA or RAD induced caspase-9 activity in COLO205 cells under FIS stimulation; however, no change in caspase-9 activity was observed in FIS-, GA-, or RAD-treated COLO205 cells (Figure 3(c)). Bcl-2 family proteins were shown to regulate the MMP and play important roles in apoptosis. Among Bcl-2 family proteins, Bcl-2 and Bcl-XL are antiapoptotic proteins, and Bax is a proapoptotic protein. In the presence of GA or RAD treatment, both Bcl-XL and Bax proteins remained unchanged in COLO205 cells under FIS stimulation; however, expression of the Bcl-2 protein was significantly reduced (Figure 4). Decreased Bcl-2 protein by RAD but not GA alone was observed, and similar results were obtained from three independent experiments. These data suggest that disruption of the MMP in accordance with induction of caspase-9 activation and a decrease in Bcl-2 protein expression was involved in GA and RAD enhancement of FIS-induced apoptosis of COLO205 cells.

### 3.4. GA Downregulates the p53 Protein at a Posttranscriptional Level in FIS-Treated COLO205 Cells

**Figure 5:**
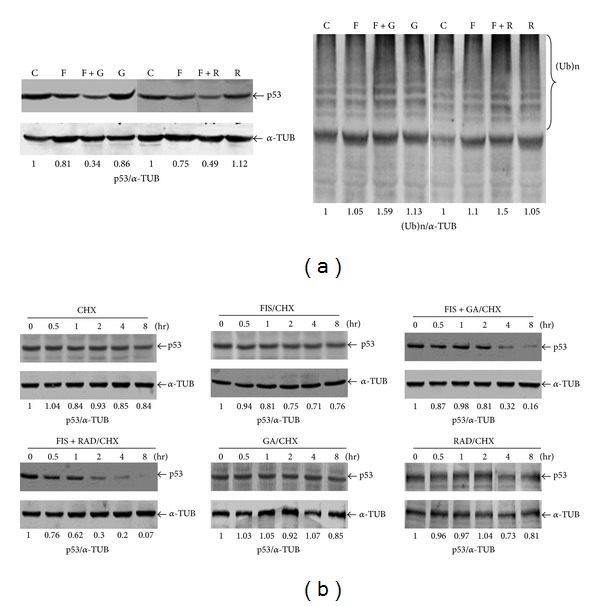
Decreased p53 and its stability, and increased ubiquitination in fisetin (FIS)+geldanamycin (GA)- or FIS+radicicol (RAD)-treated COLO205 cells. (a) Reduction of the p53 protein level and induction of ubiquitin-tagged proteins (Ub) in FIS+GA- or FIS+RAD-treated COLO205 cells. Cells were treated with FIS (60 *μ*M), GA (2 *μ*M), RAD (5 *μ*M), or their combinations for 4 h, and expressions of p53 and Ub-tagged proteins were detected by Western blotting. Intensities of (Ub)n and the p53 protein were quantified, and the ratio of p53/*α*-TUB and (Ub)n/*α*-TUB in the respective control group was described as 1. (b) GA and RAD decreased p53 protein stability in FIS-treated COLO205 cells. Cells were treated with cycloheximide (CHX; 1 *μ*g/mL) for 1 h, followed by the addition of FIS (FIS/CHX), FIS+GA (FIS+GA/CHX), FIS+RAD (FIS+RAD/CHX), GA (GA/CHX), or RAD (RAD/CHX) for different times (0, 0.5, 1, 2, 4, and 8 h). At the various time points, expressions of p53 and the *α*-tubulin protein were detected by western blotting using specific antibodies. Intensities of (Ub)n and the p53 protein were quantified, and the ratio of p53/*α*-TUB in the respective control group was described as 1. Data were repeated at least three times, and similar results were obtained.

GA's induction of apoptosis that depends on regulation by p53 was previously reported; therefore, we investigated changes in the p53 protein with and without GA or RAD in FIS-treated COLO205 cells. As shown in Figure 5(a), a significant decrease in the p53 protein was detected in FIS+GA- or FIS+RAD-treated COLO205 cells, compared to that in control, FIS-, GA-, and RAD-treated cells. Additionally, increases in ubiquitin-targeted proteins were detected in FIS+GA- or FIS+RAD-treated COLO205 cells. This indicates that the reduction in the p53 protein by FIS+GA or FIS+RAD was probably due to protein degradation. To determine whether this was the case, we measured the half-life of the p53 protein by treating cells with FIS, GA, RAD, and cycloheximide (CHX), an inhibitor of protein synthesis. As shown in Figure 5(b), the p53 protein in COLO205 cells is a very stable protein that exhibits a long half-life, and the p53 protein decreased to 84% of the initial amount after 8 h of CHX treatment. When COLO205 cells were treated with FIS/CHX, GA/CHX, or RAD/CHX, the stability of the p53 protein did not change, compared to that in CHX-treated COLO205 cells. However, following cotreatment of FIS+GA or FIS+RAD with CHX, the preexisting p53 protein markedly decreased, indicating a protein half-life of around 2 h. This finding indicates that GA decreases p53 protein in FIS-treated COLO205 cells by inducing its degradation.

## 4. Discussion

According to a systematic review of cohort studies and randomized controlled trials (RCTs), there is insufficient and conflicting evidence regarding the prevention of colorectal neoplasms by flavonoids [15, 16]. The most acceptable reason is that intake of flavonoids at an undetermined dose provides convincing clinical evidence of an anticancer potential [17–19]. However, our investigations disclosed a molecular basis for the complementary treatment of human colon cancer with a combination of flavonoids and targeted therapy. The main finding of the present study was that HSP90 inhibitors increased the cytotoxic effect of FIS in colonic carcinoma cells through inducing apoptosis. Induction of caspase-3 activation and reduction of the Bcl-2 protein in accordance with a decreased MMP and decreased p53 protein were detected in COLO205 cells treated with the combination of HSP90 inhibitors and FIS. A strategy to treat colon carcinoma with a combination of FIS and HSP90 inhibitors is suggested.

Caspases are a family of proteases that are the principal executioners of apoptosis, and their cleavage and subsequent activation are considered primary hallmarks of apoptosis. Caspase-3 is a critical executioner of apoptosis by cleavage of several essential cellular proteins such as PARP and D4-GDI. Apoptosis induction through activation of caspase activity by FIS was reported in several previous studies. Lim et al. [7] and Yu et al. [20] reported that FIS induced apoptosis and cleavage of caspases in HCT-116 colorectal carcinoma cells. Ying et al. [21] reported that FIS induced apoptosis in HeLa human cervical cancer cells through activation of a caspase-3- and -8-dependent pathway. FIS causes apoptosis in human prostate cancer LNCaP cells through activation of caspases-3, -8, and -9. Among HSP90 inhibitors, GA and RAD are prototypes of 17-N-allylamino-17-demethoxygeldanamycin (17-AAG). Concomitant use of such agents with chemotherapy was suggested to be a potential treatment for cancer [22, 23]. For example, 17-AAG abolished Akt activation and potentiated the mammalian target of rapamycin (mTOR) inhibitor, rapamycin, in breast cancer cells [24]. A combination of 17-AAG and carboplatin remarkably inhibited the growth of human ovarian cancer [25]. In our study, HSP90 inhibitors showed enhancement of the expression of apoptotic proteins in COLO205 cells under FIS stimulation. Caspase-3 activity significantly increased after concurrent treatment with HSP90 inhibitors and FIS, and the effect was diminished by adding a caspase-3 peptidyl inhibitor (Ac-DEVD-FMK). These findings show that activation of caspase cascades contributes to HSP90- and FIS-induced apoptosis in colon carcinoma cells.

The p53 protein regulates several important cellular functions, including apoptosis, cell-cycle progression, and DNA repair. p53 levels are mainly regulated at the posttranslational level by the ubiquitin-proteasome pathway, and wild-type (WT) p53 was shown to interact with HSP90 [26, 27]. The extended interaction of WT p53 with HSP90 appears to protect the p53 protein from proteolytic degradation, leading to a prolonged half-life. However, the more stable associations of p53 mutants with HSP90 leading to their misfolded conformations were reported. In the present study, the HSP90 inhibitors, GA and RAD, reduced the expression of p53 with an increase in ubiquitination activity in FIS-treated COLO205 cells. Analysis of the p53 half-life showed that the stability of the p53 protein decreased after adding FIS+GA or FIS+RAD. This indicates that enhancement of FIS-induced apoptosis by GA and RAD is mediated by disruption of p53 stability and a reduction in its half-life. Several studies indicated that COLO205 cells express WT p53 [28, 29]. These data suggest that the HSP90 inhibitors, GA and RAD, might enhance the degradation of the WT p53 protein via stimulation of protein ubiquitination to promote apoptosis in COLO205 cells.

HSP90 is involved in the establishment of cancer, and it is overexpressed in various tumors including colon, ovarian, endometrial, gastric, and pancreatic carcinomas [30, 31]. Recent data showed that HSP90 plays an essential role in facilitating the malignant transformation of tumors, is closely related to the increased proliferative potential of cancer cells, and permits tumor cells to escape apoptosis. The oncogenic effects of HSP90 provide an attractive target for treating cancer, and several HSP90 inhibitors, such as GA and RAD, were developed for in vitro and in vivo studies. Our previous study indicated that GA and RAD potentiate apoptosis in amyloid *β*-treated cerebral blood cells [32]. Misso et al. reported that the combination of tipifarnib with GA induced apoptosis in advanced head and neck squamous cell cancer [33]. Restall and Lorimer showed that GA and RAD can induce premature senescence via apoptosis in small-cell lung cancer cells [34]. Consistent with these data, GA and RAD produced enhanced FIS-induced cell death via apoptosis in COLO205 colon carcinoma cells. This suggests that a combination of HSP90 inhibitors with FIS could be a suitable therapeutic strategy for treating colon carcinoma. In intrinsic apoptotic pathway via disrupting mitochondrial functions, activation of caspase-9 is regulated by members of Bcl-2 family. It is known that decreased antiapoptotic Bcl-2-family proteins such as Bcl-2 and increased proapoptotic Bcl-2-family proteins such as Bax lead to apoptosis. Our results showed that FIS+GA and FIS+RAD can cause a decrease in Bcl-2 protein with an increase in the cleavage of caspase-9 protein, indicating that FIS+GA and FIS+RAD can activate intrinsic mitochondrial apoptotic pathway. An interesting observation is that RAD showed inhibitory effect on Bcl-2 protein expression without affecting caspase-9 activity and mitochondrial membrane potential in COLO205 cells. Yang et al. reported that RAD and GA reduced viability of ovarian carcinoma cells with a decrease in Bcl-2 protein level [35]. Further studies are needed to elucidate the mechanism of RAD-inhibited Bcl-2 protein level in cancer cells.

Taken together, our data reveal that the HSP90 inhibitors, GA and RAD, enhanced the cytotoxicity of FIS via activation of a mitochondria-dependent caspase-3 cascade and accelerated the degradation of the p53 protein. The mechanism of GA and RAD regulated p53-chaperon interactions, and the influence of GA and RAD on HSP90's chaperon function requires further investigation. Our findings highlight the potential of GA and RAD in combination with anticancer drugs, such as FIS, for further in vivo treatment of colon carcinoma.

## Abbreviations

FIS: Fisetin
HSP90: Heat shock protein 90
GA: Geldanamycin
RAD: Radicicol
BCIP: 5-Bromo-4-chloro-3-indolyl phosphate
Bcl-2: B-cell lymphoma 2
Ub: Ubiquitin
DCFH-DA: 2′,7′-Dichlorofluorescein diacetate
MMP: Mitochondrial membrane potential
MTT: 3-(4,5-Dimethylthiazol)-2-yl-2,5-diphenyltetrazolium bromide
NBT: Nitroblue tetrazolium
PARP: Poly(ADP-ribose)polymerase
Casp 3: Caspase-3
Casp 9: Caspase-9.
